# Intramammary treatment using allogeneic pure platelet-rich plasma in cows with subclinical mastitis caused by Gram-positive bacteria

**DOI:** 10.1038/s41598-021-03067-4

**Published:** 2021-12-09

**Authors:** Paulo C. Duque-Madrid, Juan Velasco-Bolaños, Alejandro Ceballos-Márquez, Catalina López, Jorge U. Carmona

**Affiliations:** 1 Grupo de Investigación en Calidad de Leche y Epidemiología Veterinaria (CLEV), Departamento de Producción Agropecuaria, Universidad de Caldas, Calle 65 No 26-10, Manizales, Colombia; 2 Grupo de Investigación Terapia Regenerativa, Departamento de Salud Animal, Universidad de Caldas, Calle 65 No 26-10, Manizales, 170004 Colombia

**Keywords:** Regenerative medicine, Cytokines, Infection, Infectious diseases

## Abstract

The aims of the study were (1) to compare the cure risk of intramammary treatment of pure platelet rich plasma (P-PRP) or cefquinome sulfate (CS) in cows with subclinical mastitis (SCM) caused by Gram-positive bacteria, evaluated via somatic cell count (SCC) and the microbiological analysis of milk; (2) to compare the inflammatory/anti-inflammatory response of mammary gland to both treatments through the analyses of interleukins (IL), interferon gamma (IFN-γ), and tumour necrosis factor alpha (TNF-α) in milk. A non-inferiority randomized clinical trial was conducted. The null hypothesis was that cure risk in the experimental group (P-PRP) was inferior to the reference group (CS). A total of 103 cows were selected according to SCC and presence of Gram-positive bacteria, 49 cows were treated with CS and 54 cows were treated with P-PRP after determination of its cellular and molecular quality control. Cure was assessed by milk analyses at day 21 and 22 after treatment. Cows that remained with SCM were retreated at day 26, and cure assessed at day 47 and 48. Overall, bacteriological cure was observed in 16 cows (30%) of the P-PRP group, and 35 cows (71%) in CS group. *Staphylococcus aureus* cure risk was higher in CS group, but inconclusive for *Streptococcus* spp. The mean SCC increased in relation to time only in the P-PRP group. A direct relation between time and treatment for IL-1, IL-2, and IL-6 was observed, while no differences were observed for IL-4. Furthermore, IL-1 and IL-2 increased in cows treated twice in both groups. IL-8, IFN-γ, and TNF-α showed a significant interaction between time and treatment. IFN-γ concentration was lower in the P-PRP group compared to the CS on days 0 and 22. Leukocyte counts were lower in P-PRP when compared to whole blood. TGF-β1 and PF4 concentrations were higher in platelet lysates in comparison to P-PRGS and plasma. Moreover, PDGF-BB concentration was significantly higher in platelet lysates in comparison to plasma. Results obtained in this study demonstrate that SCM treated with PRP showed a lower rate of bacteriologic cure when compared to animals treated with CS.

## Introduction

Subclinical mastitis (SCM) is an important cause of economic losses in the global dairy cattle industry^1–3^. This disease is characterized by the absence of clinical signs of mammary gland inflammation, such as heat, redness, pain, and edema. Subclinical mastitis is typically associated with high somatic cell count (SCC), with mammary pathogens are commonly being the infective cause^4^. Udder health programs routinely include monitoring individual SCC evaluation to detect cows that exhibit increased SCC, that could be suggestive of SCM^5^, often due to pathogenic bacteria in milk^6–8^.

The most common bacterial isolates from cows with SCM are represented by Gram-positive pathogens (60% or more), such as *Staphylococcus aureus*, *Streptococcus uberis*, *Streptococcus dysgalactiae*, *Streptococcus agalactiae,* and coagulase-negative staphylococci (CNS)^7,9–11^. The latter, consisting of over 45 different species and subspecies^11,12^. Currently, the cornerstone treatment for SCM includes the use of antibiotic preparations during lactation and at drying-off^11–13^. However, the use of antibiotics for the treatment of this disease remains controversial due to the presence of antibiotic residuals in milk, the development of bacterial resistance to antimicrobials, and low cure rate associated with SCM, amongst others^13,14^. Due to aforementioned it remains necessary to research and develop alternative therapeutic strategies for SCM treatment^13^.

In line with this, platelet concentrates (PCs) or related hemoderivatives may represent an alternative therapy for cows with SCM. According to the classification of Dohan Ehrenfest et al.^15^, liquid PCs can be classified into two groups: (1) pure platelet-rich plasma (P-PRP) products, which are hemoderivatives without leukocytes or with negligible concentrations of these cells and a low-density fibrin network after activation and; (2) leukocyte-PRP (L-PRP) products, which are hemoderivatives with leukocytes and a low-density fibrin network after activation. Once these hemoderivatives are activated with calcium salts or thrombin, they are transformed into a platelet-rich gel (PRG). Thus, the PRG from L-PRP is termed L-PRG, and the PRG from P-PRP is called P-PRG^15,16^.

Platelet-related hemoderivatives, such as P-PRP, are an important source of growth factors (GFs) including transforming growth factor beta 1 (TGF-β_1_) and platelet-derived growth factor (PDGF), among other polypeptides necessary to induce wound healing and reduce inflammation^17^. Furthermore, some in vitro^18–23^ and in vivo^24,25^ studies have described an important bacteriostatic effect of this substance against several Gram-positive and Gram-negative bacteria, possibly through the plasma complement, [i.e., C3 fraction (C3)]^26,27^ and some chemokines released during platelet activation, such as beta-defensin and platelet factor 4 (PF4)^28,29^. Therefore, it appears that the bacteriostatic effect described for several PCs is not related to the concentrations of white blood cells (WBCs) in such hemoderivatives^18,21,22,30^.

In a previous study, Italian researchers discovered that an allogeneic platelet-related hemoderivative known as platelet lysate was useful in treating cows with both acute and chronic clinical mastitis (CM) caused by Gram-positive and Gram-negative bacteria^31^. The results obtained in that study were encouraging, as platelet lysates produced a ~ 67% reduction in SCC in cows with acute CM, and a 53% reduction in animals with chronic CM in comparison to treatment with antibiotics (52.5% and 15.4%, respectively) and a combination of both platelet lysate and antibiotics (90.6% and 66.7%, respectively). However, the risk of microbiological cure was not established. Furthermore, although the platelet lysate was obtained through a frozen-and-thawed procedure from a PRP preparation with a final platelet concentration of 1 × 10^6^/μl, the authors did not report the WBC or GF concentration of this substance^31^.

To our knowledge, there are no published reports regarding the therapeutic use of P-PRP in dairy cows for treatment of SCM caused by Gram-positive bacteria. Furthermore, there are no data related to immunologic response to PRP preparations of the mammary gland in cows with SCM. Therefore, we hypothesized that PRP would be a viable alternative for treating SCM in dairy cows and would be efficacious as intramammary antibiotics in a non-inferiority trial. Moreover, P-PRP would produce an improved anti-inflammatory profile in the milk of treated cows when compared to those treated with intramammary antibiotics.

The aims of the present study were (1) to compare the cure risk between a group of cows treated with an experimental product (P-PRP group) and a group treated with a reference product (CS group) through SCC and the microbiological analysis of milk; and (2) to compare the inflammatory and anti-inflammatory response of the mammary gland in both groups (P-PRP and CS groups) through the analyses of interleukins, and the tumor necrosis factor alpha (TNF-α) in milk.

## Materials and methods

The Committee for Animal Experimentation of Universidad de Caldas approved all the protocols described in this section before starting the trial. All the methods performed in this study were carried out in accordance with The Guide for the Care and Use of Laboratory Animals of the National Research Council and the Institute for Laboratory Animal Research of USA and the Colombian Act of Animal Welfare. Furthermore, this study was carried out in compliance with the ARRIVE guidelines. However, the animals evaluated in this study were Holstein cows from commercial herds. The cows never were slaughtered in the present study for research purposes.

### Study design

The study design for this treatment comparison was a non-inferiority randomized clinical trial following a previously described methodology^32^. The null hypothesis was that the treatment with the experimental treatment (P-PRP) was inferior to the reference product (cefquinome sulfate, CS) administered to the reference group. The alternative hypothesis was that the experimental treatment is not inferior by more than the predefined margin (− *Δ*):

1 $${\text{H}}_{0} : \, \left[ {R_{{{\text{cure}}}} \left( {{\text{CS}}} \right) \, - R_{{{\text{cure}}}} \left( {{\text{P}} - {\text{PRP}}} \right)} \right] \, \le \, - \Delta ,$$

2 $${\text{H}}_{{\text{a}}} : \, \left[ {R_{{{\text{cure}}}} \left( {{\text{CS}}} \right) \, - R_{{{\text{cure}}}} \left( {{\text{P}} - {\text{PRP}}} \right)} \right] \, > \, - \Delta ,$$

where *R*_cure_ is the cure risk and *Δ* is the non-inferiority margin. Results were interpreted according to the principles for a non-inferiority trial^32,33^. Thus, rejecting H_0_ results in acceptance of H_a_, indicating that P-PRP is non-inferior to CS.

### Sample size

The sample size calculation was based on previous results using platelet lysates for the treatment of CM, assuming that PRP cure risk was approximately 35%^31^, and a significance level of 5% and power of 80% were selected. Selection of *Δ* is often based on the results of negative controlled trials, with *Δ* being no more than half the effect expected from a superiority study^34^. However, to our knowledge, there are no negative controlled studies conducted to treat SCM in cows using P-PRP. Therefore, the *Δ* was chosen as half of the effect of the results reported by Lange-Consiglio et al. (*Δ* = 17.5%)^31^. If there is a true difference in favor of the experimental treatment of 5%, then 108 (i.e., 54 cows per group) cows with SCM are required to be 80% certain that the upper limit of a one-sided 95% confidence interval (CI) (or equivalently, a 90% two-sided CI) will exclude a difference in favor of the CS group of more than 17.5%.

### Dairy herds and animals

A total of 103 cows from nine commercial herds in Caldas and Risaralda provinces of Colombia were selected for the study. Cows were managed under rotational grazing systems and received supplementation with concentrates according to milk yield. Predominant pastures were a mixed of Kikuyu grass (*Pennisetum clandestinum*), Orchardgrass (*Dactylis glomerata* L.), and Yorkshire fog grass (*Holcus lanatus*). Concentrates used were commercial mixes of cereals, containing between 14 and 16% of crude protein, and approximately 2.9 Mcal ME/kg of dry matter. Concentrates were fed starting at three weeks before calving (2 kg/cow/day), and at a rate of 1 kg per 4 kg of milk yield after calving. Mineral supplements and water were available ad libitum.

### Inclusion criteria

Cows for the trial were eligible for inclusion in the study when they were in parity 1 to 5, did not have blind mammary quarters, had experienced no cases of CM in the current lactation, and had received no treatment with antibiotics for any other disease in the last 30 days.

### Bacteriological analysis and intramammary infection declaration

Two individual composite milk samples from each cow were aseptically collected before milking at the first visit to the herd in order to select the cows to be treated for SCM. Milk samples were then refrigerated and submitted to our laboratory for analysis. One sample was used for the analysis of SCC using an automated cell counter (Fossomatic, Foss, Hillerød, Denmark). The SCC results were expressed as the natural log (LnSCC, in thousands/mL) to normalize the data distribution.

Based on the SCC results, the second sample was used for bacteriological and polypeptide analyses whenever SCC was ≥ 100,000 cells/mL in primiparous cows and ≥ 200,000 cells/mL in multiparous cows^5^. Microbiological analyses were performed according to protocols established by the National Mastitis Council^35^. Briefly, milk samples were streaked onto agar plates containing 5% sheep blood and 0.1% esculin. Agar plates were incubated at 37.5 °C and visually inspected at 24 h and 48 h for bacterial growth. Morphology and Gram staining were used for initial identification. Then a catalase test was used to differentiate staphylococci from streptococci. The coagulase test was performed on catalase-positive cocci to confirm the presence of *Staphylococcus aureus.* Gram-positive and catalase-negative cocci were differentiated by their reaction to the hydrolysis of esculin under ultraviolet light. The Christie, Atkins, Munch-Petersen (CAMP) test and esculin reaction were used to differentiate *Streptococcus agalactiae* and *Strep. dysgalactiae*, while esculin reaction and culture in enterococcosel (Becton–Dickinson, Durham, NC, USA) agar were used for the identification *Strep. uberis*. Their morphology and Gram staining were used to identify *Corynebacterium* spp. Growth in MacConckey agar and biochemical tests such as citrate, indole, oxidase and motility tests were used to identify coliforms. Cultures that presented more than two bacterial species were considered contaminated and not informative of intramammary infection (IMI).

Intramammary infection was declared when a composite milk sample presented a SCC higher than the cut points established and the microbiologic culture was positive for any major Gram-positive mammary pathogen (*Staph. aureus, Strep. agalactiae, Strep. uberis* and *Strep. dysgalactiae*). All culture-positive cows meeting the SCC selection criteria were treated.

### Treatment protocols

Cows with SCM were randomly allocated to one of two treatment groups using random function in Excel (Microsoft Corp., Redmond, WA, USA). A total of 54 cows diagnosed were treated with P-PRP, while 49 cows were treated with CS. The initial treatment was administered 4 days after the first milk sampling. Each quarter of each cow of both P-PRP and CS groups, were treated post-milking and after teat disinfection with the administration of three tubes at 12 h intervals. The P-PRP group was treated with an intramammary infusion of 10 mL of P-PRP activated with calcium gluconate (9.3 mg/mL) (Ropsohn Therapeutics, Bogota DC, Colombia) in a proportion of 1:10; and CS group received the on-label treatment, which consist of 75 mg of CS during three consecutive milkings (Cobactan LC, Merck Animal Health, Madison, NJ, USA).

### Blood procurement and P-PRP preparation

The P-PRP used in this study was obtained from four *Blanco Orejinegro* heifers aged ranged between 12 and 18 months, and mean body weight of 300 ± 20 kg. Animals were sedated with xylazine (0.01 mg/kg, IM) and restrained in right lateral recumbence on a surgical table. Then, the skin of the left lateral jugular vein was aseptically prepared for blood extraction using an iodine povidone scrub and cleared with ethyl alcohol. A small area of the skin over the jugular vein in the central region of the neck was desensitized by the injection of 2 mL of lidocaine (2%). A skin incision of 3 mm was performed with a sterile surgical blade. Then, a 14 G catheter was placed into the jugular vein, and a heparin rubber cap was placed on the catheter to avoid the risk of bleeding.

The blood of each donor was obtained by coupling the needle of a double transfusion bag to the rubber cap. The blood was extracted to the transfusion bag, which was constantly and smoothly shaken to mix the blood and the anticoagulant. A total of three bags of 500 mL were obtained from each heifer during each round of blood extraction. All heifers were closely monitored for anemia (through a weekly complete cell blood count) and any other health issues. All animals were bled every two weeks until treatments in the P-PRP group were complete.

Following extraction, the blood bags were immediately centrifuged in a stationary centrifuge (RotoSilenta 630 RS. Hettich, Tuttlingen, Germany) at 1600*g* for 8 min. Then, the plasma fraction (P-PRP), including the buffy coat of each blood bag, was transferred to a separated plasma bag. The P-PRP was gently homogenized and packed into 10 mL syringes in a laminar flow cabinet. The free extreme of each P-PRP syringe was protected with a plastic sterile cap. An additional sterile reaction tube containing 1 mL of calcium gluconate was packed into the 10 mL syringe containing the P-PRP in a sterile plastic bag. Calcium gluconate was added to the P-PRP immediately before the intramammary infusion. This substance was used to induce platelet activation and subsequent growth factor release from P-PRP.

### Cellular and polypeptide quality control of P-PRP

The platelet and leukocyte counts as well as TGF-β_1_, PDGF, PF4, and C3 concentrations in whole blood and P-PRG supernatants (P-PRGS) were compared to demonstrate the enrichment of the hemoderivative experimentally evaluated in the present study as follows.

Thirty P-PRP syringes of 10 mL each were randomly chosen for a blood count using automated equipment (Celltacα MEK-6450. Nihon Kohden, Tokyo, Japan). Samples were also analyzed by Enzyme-Linked ImmunoSorbent Assay (ELISA) to determine the concentration of polypeptides released from P-PRGS (i.e., TGF-β_1_, PDGF-BB, PF4, and C3). P-PRP was incubated with calcium gluconate in a 10:1 dilution for 6 h. The same molecules were measured in plasma (i.e., a negative control) obtained using the same anticoagulant but centrifuged at a higher speed (5000*g*). We used P-PRP samples incubated with a 0.5% solution of a non-ionic detergent (platelet lysates) (Triton X100, PanReacAppliChem, Barcelona, Spain) in a 10:1 dilution for 15 min as a positive control of polypeptide release, because Triton X100 induces membrane cell damage and complete release of GFs and cytokines from platelets and leukocytes.

### Post-admission sampling

Once cows were treated, composite milk samples from both groups were collected at days 21 and 22, and each sample was analyzed for microbiological diagnosis and SCC. Procedures for SCC and microbiology were previously described in “Bacteriological analysis and intramammary infection declaration” section. The treatment was repeated in cows that remained positive for major Gram-positive bacteria, and they received the same product used for the first treatment 4 days after collecting the second milk sample. A final milk sampling was conducted on all refractory cases 21 and 22 days after the repeated treatment (i.e., days 47 and 48). Individual cow SCC, and pre- and post-treatment concentration of cytokines (IL-1, IL-2, IL-4, IL-6, IL-8, IFN-γ, and TNF-α) were generated for each milk sample. No more than two treatment attempts per cow were used in the present study.

### Concentration of cytokines in hemoderivatives and milk

Both hemoderivatives and milk samples were thawed at room temperature and used for ELISA analysis without further centrifugation. Mediators were analyzed in hemoderivatives and whole milk using commercial ELISA development kits from R&D Systems (Minneapolis, MN, USA) with the exception of IL-1, IL-4, IL-8 and C3. PDGF-BB (Human PDGF-BB DuoSet, DY220), TGF-β_1_ (Human TGF-β1 DuoSet, DY240E), and PF4 (Human CXCL4/PF4 DuoSet, DY795) were determined using human antibodies. Notably, TGF-β_1_ shares a high homology between these proteins in humans and cattle^36^. Furthermore, human PDGF-BB antibody has been also used to measure the same bovine protein in other studies^37,38^, while PF4 presents a high homology between humans and ruminants^39^. Interleukin 1 (Bovine IL-1β ELISA Reagent Kit, Thermo Fisher Scientific Inc., Waltham, MA, USA), IL-2 (Bovine IL-2 DuoSet ELISA. R&D Systems, Minneapolis, MN, USA), IL-4 (IL-4 ELISA Development Kit. Mabtech AB, Nacka Strand, Sweden), IL-6 (Bovine IL-6 DuoSet ELISA. R&D Systems, Minneapolis, MN, USA), IL-8 (IL-8 ELISA Development Kit. Mabtech AB, Nacka Strand, Sweden), IFN-γ (Bovine IFN-gamma DuoSet. R&D Systems, Minneapolis, MN, USA), TNF-α (Bovine TNF-alpha DuoSet ELISA, R&D Systems, Minneapolis, MN, USA), and C3 (Bovine Complement Component 3 ELISA Kit. MyBioSourceInc., San Diego, CA, USA) were assayed with bovine-specific antibodies. The standards provided for each ELISA kit were used in preparing each standard curve according to the manufacturers’ instructions. Readings were performed at 450 nm. In general, the inter- and intra-assay coefficients of variation for the various ELISA kits were between 2 and 5%.

### Primary and secondary outcomes

Cure was established in cows that were infected at the beginning of the study and where the organism present was not isolated in the subsequent two post-treatment sample.

Cure was defined at the cow level, and cure risk was statistically assessed for the initial treatment (samples at 21 and 22 days) and treatment of refractory cases samples (samples at 47 and 48 days) independently.

A reduction in SCC was defined as a decrease in SCC for the two post-treatment samples compared to the first value at the start of the study. Changes in milk cytokine concentrations were also used as a criteria for cure and the evaluation of mammary gland inflammation.

### Statistical analysis

Platelet and leukocyte counts in whole blood and P-PRP were compared using a Mann–Whitney U Test. The concentrations of TGF-β_1_, PDGF-BB, PF4, and C3 in hemoderivatives (plasma, platelet lysates, and P-PRGS) were analyzed by one-way ANOVA followed, if necessary, by a Tukey test.

Statistical analysis of the various outcomes was performed using linear and logistic regression models according to the response variable^40^. The main predictor was the treatment group (P-PRP and CS). The analyses considered covariates such as herd, cow parity, and the natural log of SCC (LnSCC) before treatment. The logistic regression model that was used for bacteriological cure was:

3 $${\text{Logit }}\left( {p\,{\text{of}}\,{\text{ cure}} = {1}} \right) \, = {\text{ intercept }} + {\text{ treatment }} + {\text{ herd }} + {\text{ parity }} + {\text{ LnSCC,}}$$

where cure is the bacteriological cure for major Gram-positive pathogens, treatment is a binary variable indicating either P-PRP or CS, herd is a set of indicator variables for dairy herd, parity is parity of the enrolled cow, and LnSCC is the LnSCC value before treatment. Logistic regression models were also used to determine the probability of cure of *Staph. aureus* and *Streptococcus* spp. as outcome variables individually. *Streptococcus* spp. was a group comprised of the major pathogens of streptococci genera isolated in the study i.e., *Strep. agalactiae, Strep uberis, and Strep dysgalactiae*.

The non-inferiority trial was obtained by a two-proportion test and based on the upper limit of the 95% CI of the risk of cure, the noninferiority was claimed if the difference in the test was smaller than the *Δ*^41^*.*

The statistical analyses of LnSCC and milk concentration of IL-1, IL-2, IL-4, IL-6, IL-8, IFN-γ, and TNF-α results were performed using linear regression. The main predictor was treatment, and the fixed effects of covariates such as herd, parity, and LnSCC before treatment were also considered. The linear regression model was:

4 $$y = {\text{ intercept }} + {\text{ treatment }} + {\text{ herd }} + {\text{ parity }} + {\text{ LnSCC }} + {\text{ error,}}$$

where *y* corresponds to LnSCC after treatment on day 21 and day 47 for cows treated one or two times, respectively, and the milk concentration of IL-1, IL-2, IL-4, IL-6, IL-8, IFN-γ, and TNF-α, which were measured only at days 0, 21, and 47, where day 0 was the reference value to compare against day 21 and 47 results. The other predictors were previously described.

The analyses were conducted in Stata 14.1 using the commands ‘ranksum’, ‘anova’ ‘*prtest*’, ‘*logit*’ and ‘*reg*’ (Stata Corp. College Station, TX, USA). A *P*-value of < 0.05 was accepted as statistically significant for all tests.

## Results

### Cellular and molecular P-PRP quality control

**Figure 1:**
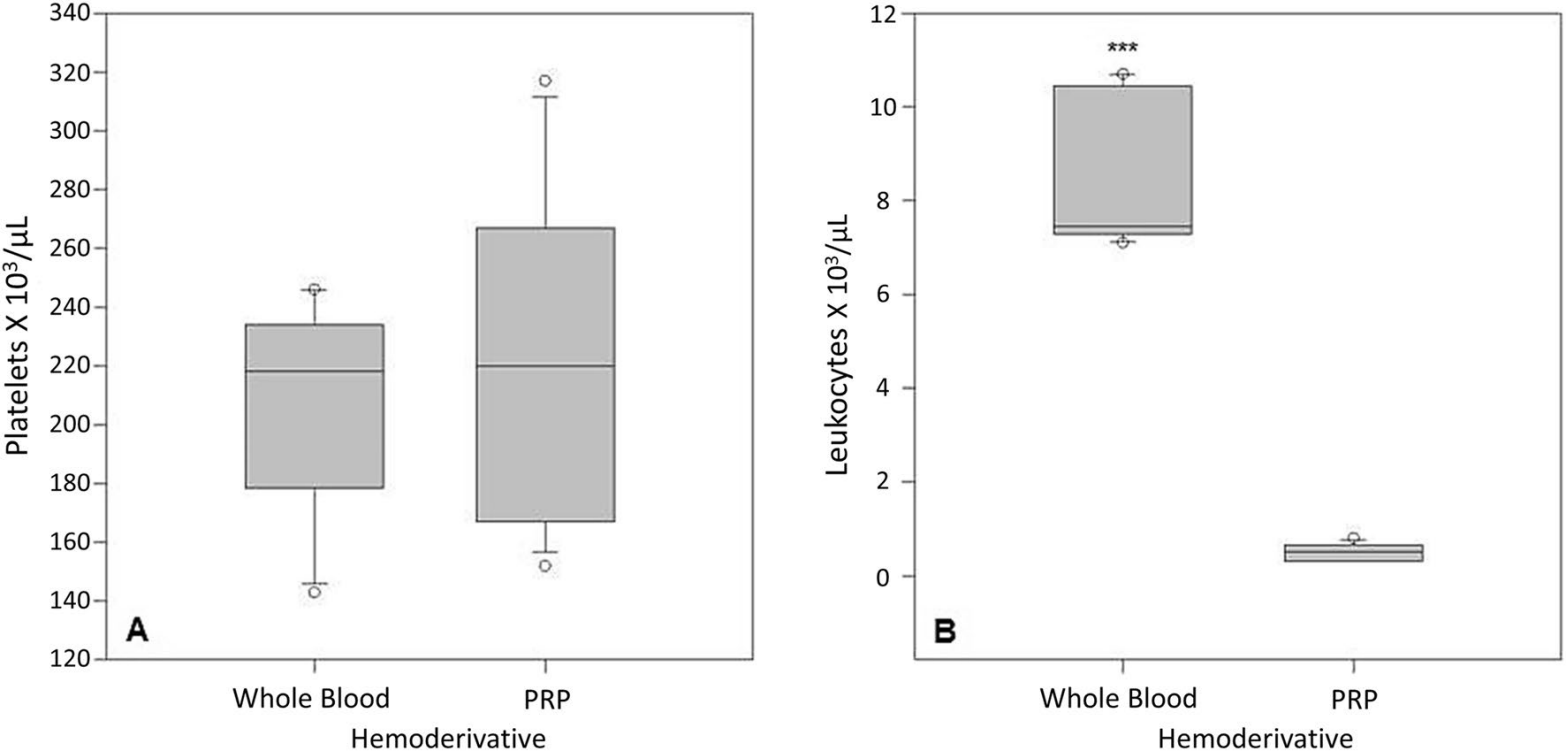
Box plot representation of the median counts of platelets/μL (**A**) and leukocytes/μL (**B**) in hemoderivatives. *PRP* pure platelet-rich plasma. ***Significant difference (P < 0.0001).

**Figure 2:**
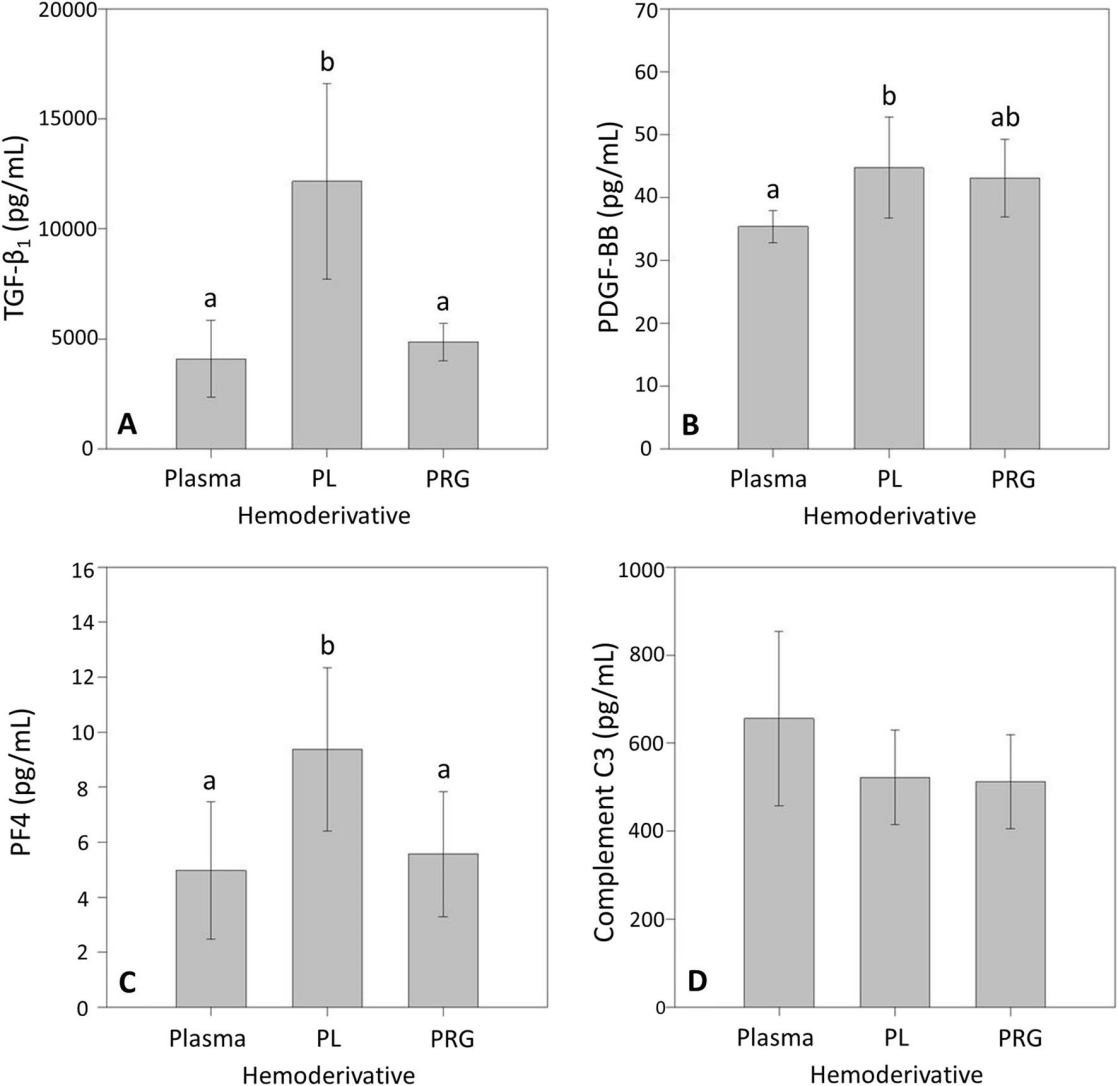
Mean and 95% confidence interval of the concentration (pg/mL) of (**A**) transforming growth factor beta 1 (TGF-β_1_), (**B**) platelet-derived growth factor BB (PDGF-BB), (**C**) platelet factor 4 (PF4), and (**D**) complement C3 (C3) in hemoderivatives. Different letters represent significant differences (at P < 0.05) for each independent protein between hemoderivatives. *PL* platelet lysate, *PRG* pure platelet-rich gel supernatant.

Platelet counts were not statistically different between whole blood and P-PRP (Fig. 1A). However, leukocyte counts were significantly (P < 0.01) lower in P-PRP when compared to whole blood (Fig. 1B). The concentrations of TGF-β_1_ and PF4 were significantly (P < 0.01) higher in platelet lysates (positive control) in comparison to P-PRGS and plasma (Fig. 2A,C). PDGF-BB concentration was significantly higher in platelet lysates in comparison to plasma, but they were not statistically different between plasma and P-PRGS (Fig. 2B). On the other hand, C3 concentrations were similar between the evaluated hemoderivatives (Fig. 2D).

### Intramammary infections and bacteriological cure

**Figure 3:**
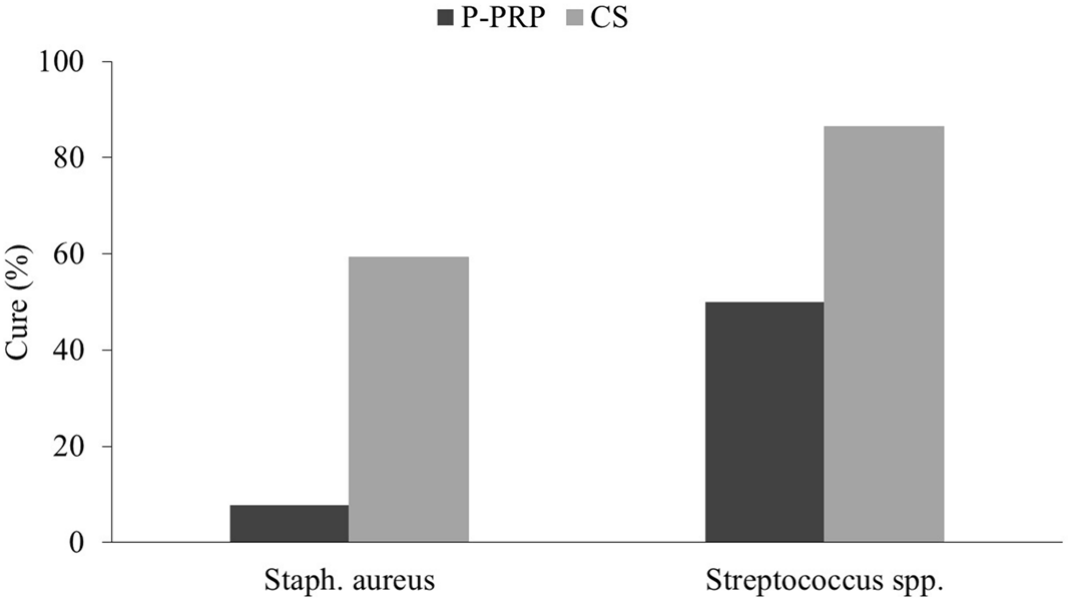
Overall bacteriological cure rate among cows with subclinical mastitis caused by Gram-positive pathogens, which were treated with either 10 mL of intramammary P-PRP (pure platelet rich plasma) or 75 mg of intramammary CS (cefquinome sulfate) over three consecutive milkings, *Streptococcus* spp. includes only *Streptococcus uberis*, *Streptococcus agalactiae* and *Streptococcus dysgalactiae*.

**Figure 4:**
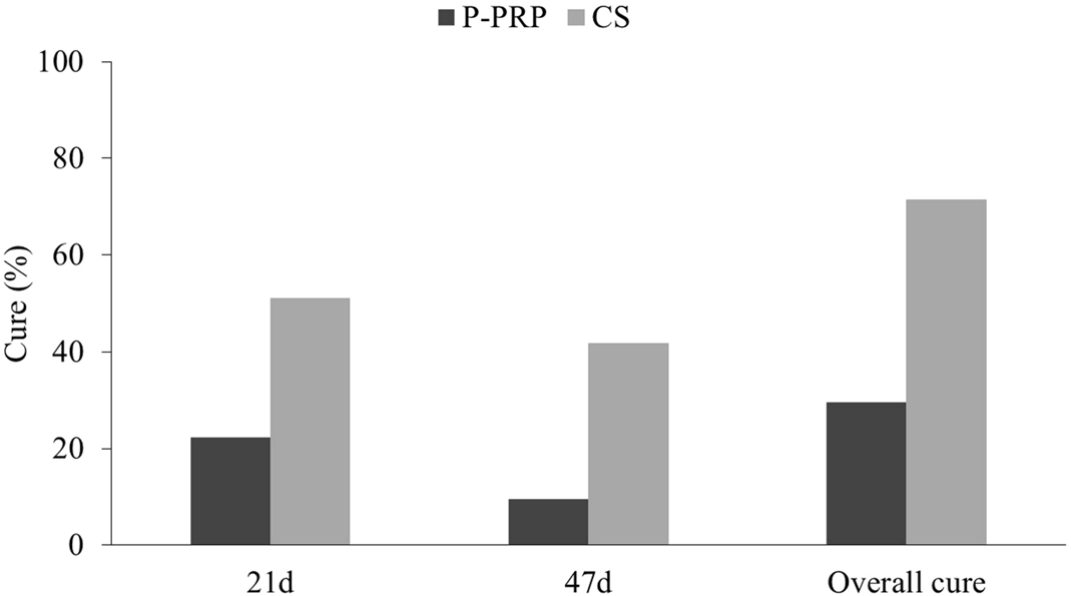
Bacteriological cure rate at 21 days and 47 days, and the overall cure rate in cows with subclinical mastitis caused by Gram-positive major pathogens following intramammary treatment with 10 mL of P-PRP (pure platelet rich plasma) or 75 mg of CS (cefquinome sulfate) over three consecutive milkings.

Enrolled farms contributed 103 cases of SCM (Table 1). Parity of the cows was between 1 and 4 in both treatment groups P-PRP and CS. The relative distribution of IMIs before the start of the study are provided in Table 2. Overall, bacteriological cure was observed in 16/54 cows (30%) of the P-PRP group, while bacteriological cure was observed in 35/49 cows in the CS group (71.0%). Figure 3 presents the distribution of bacteriological cure by initial bacteriological status and by treatment. A total of 66/103 cows (65%), 42/54 cows of the P-PRP group and 24/49 cows from CS group received two treatments because they were refractory to the first treatment (Fig. 4).

**Table 1:** Number of subclinical mastitis cases contributed per dairy herd (grouped by treatment).

Farm number	P-PRP group (n = cows)	CS group (RG) (n = cows)	Cows per farm
1	2	1	3
2	8	9	17
3	4	3	7
4	5	3	8
5	7	5	12
6	3	4	7
7	4	5	9
8	12	11	23
9	9	8	17
Total cows	54	49	103

*CS* cefquinome sulfate, *P-PRP* pure platelet rich plasma.

**Table 2:** Frequency of isolated pathogens in the 103 subclinical mastitis cases at the enrollment in the study (time zero).

Pathogen	P-PRP	CS	Total
*Staphylococcus aureus*	26	27	53
***Streptococcus *spp.**^a^	28	22	50
*Streptococcus uberis*	20	18	38
*Streptococcus agalactiae*	6	4	10
*Streptococcus dysgalactiae*	2	0	2
Total	54	49	103

*CS* cefquinome sulfate, *P-PRP* pure platelet rich plasma.

^a^Includes only *Streptococcus uberis*, *Streptococcus agalactiae* and *Streptococcus dysgalactiae*.

Logistic regression models of bacteriological cure resulted in higher cure risks for cows treated using CS compared to the P-PRP-treated cows (Table 3). The multivariable model showed a non-significant effect of parity (P = 0.50), while the effect of LnSCC at the start of the study on the probability of cure was approaching significance (P = 0.08). Therefore, a second model without the parity was analyzed. This model resulted in a *P*-value increase, hence there was no effect of LnSCC at the beginning of the trial (P = 0.11). The effect of herd was significant in both models full (P = 0.015), and reduced (P = 0.007); though, the coefficients are not shown.

**Table 3:** Coefficients and standard error (SE) of the logistic regression model for evaluating the effect of intramammary treatments (10 mL of P-PRP and 75 mg of CS for three consecutive milkings) on the probability of bacteriological cure in cows with subclinical mastitis caused by Gram-positive pathogens.

Effect	Coefficient	SE	*P*-value
**Treatment**			< 0.001
P-PRP	− 3.18	0.70	
CS	Reference		
Herd (1 to 9)	Not shown		0.007
LnSCC	0.31	0.20	0.11

*CS* cefquinome sulfate, *LnSCC* natural log of somatic cell count, *P-PRP* pure platelet rich plasma, *SE* standard error.

The coefficients of the logistic model to evaluate the effect of treatment on the cure risk for *Staph. aureus* and the *Streptococcus *spp. group are presented in Table 4.

**Table 4:** Coefficients and standard error (SE) of the logistic regression model for evaluating the effect of intramammary treatments (10 mL of P-PRP and 75 mg of CS for three consecutive milkings) on the probability of cure in cows infected with *Staphylococcus aureus* and *Streptococcus* spp. (includes only *Streptococcus uberis*, *Streptococcus agalactiae* and *Streptococcus dysgalactiae*).

Effect	Coefficient	SE	*P*-value
**Treatment for *Staph. aureus***			0.001
P-PRP	− 2.86	0.83	
CS	Reference	–	
**Treatment for *Streptococcus* spp.**			0.01
P-PRP	− 1.85	0.72	
CS	Reference	–	

*CS* cefquinome sulfate, *P-PRP* pure platelet rich plasma, *SE* standard error.

**Figure 5:**
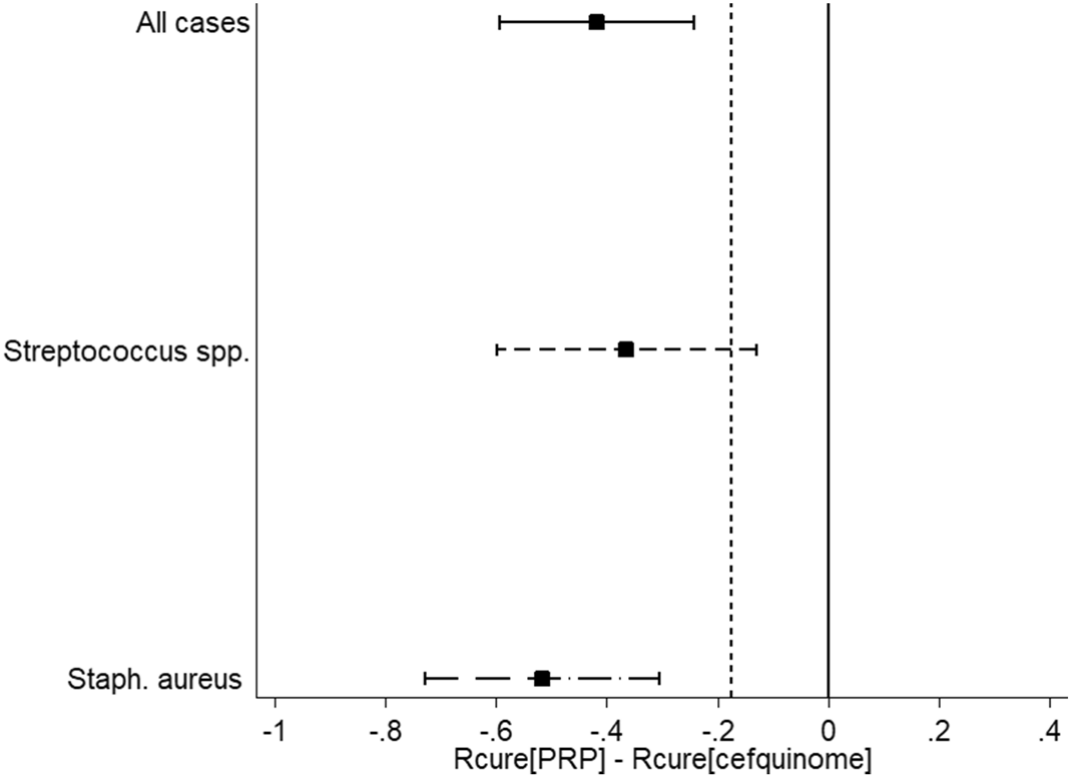
Graphical representation of the risk difference for bacteriological cure between 10 mL of P-PRP (pure platelet rich plasma) and 75 mg of CS (cefquinome sulfate) over three consecutive milkings. The black dotted vertical line represents the non-inferiority margin (*Δ*), and the area to the right of this value (− 0.175) indicates the zone of non-inferiority. The solid square is the point-estimate of difference in cure between P-PRP and CS; caps of the horizontal lines indicate 95% CI. All cases = bacteriological cure of all cases, the CI does not span both zero and the non-inferiority margin (− *Δ*), which indicates that CS is significantly better. *Streptococcus* spp. (includes only *Streptococcus uberis*, *Streptococcus agalactiae* and *Streptococcus dysgalactiae*) = bacteriological cure in *Streptococcus* spp. cases: the CI spans − *Δ* but not 0, implying that CS is better, however, non-inferiority is inconclusive. *Staph. aureus* = bacteriological cure in *Staphylococcus aureus* cases: the CI does not span both − *Δ* and zero, thus CS is significantly better.

These results demonstrate that the probability of bacteriological cure for infections caused by *Staph. aureus* in the P-PRP group was 0.06 times that for CS group. In the case of infections caused by *Streptococcus* spp*.* the probability of cure with P-PRP treatment was 0.16 times that for the CS. The cure risk for each treatment was used to calculate the risk difference [*R*_cure_(P-PRP) − *R*_cure_(CS)]. Moreover, a significant difference (P < 0.05) was observed between both treatments for all cases, where P-PRP treatment was inferior to CS. The differences in bacteriological cure at the pathogen level with their 95% CI are presented in Fig. 5.

### Somatic cell count

**Figure 6:**
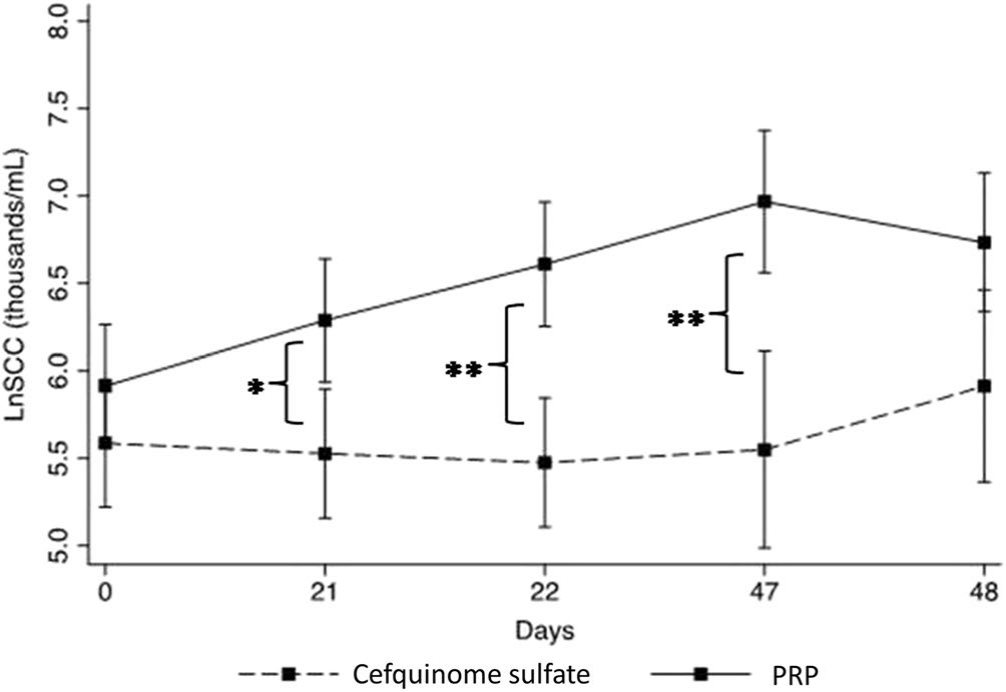
Means and confidence intervals of LnSCC (thousands/mL) for cows with subclinical mastitis that received intramammary treatment with 10 mL of P-PRP (pure platelet rich plasma) or 75 mg of CS (cefquinome sulfate) over three consecutive milkings. Zero represents the day of the treatment. The LnSCC of days 47 and 48 corresponds to cows treated twice, as they were refractory to the first treatment. * = Significantly different (P < 0.05). ** = Significantly different (P < 0.01).

A total of 425 milk samples collected at day zero (day of treatment) and day 21, 22, 47 and 48 were analyzed for SCC. No differences in LnSCC were observed at the start of the trial. The geometric mean for the P-PRP group was 370 × 10^3^ cells/mL, and 266.7 × 10^3^ cells/mL for the CS group (Fig. 6). Mean LnSCC significantly increased in the P-PRP group after the start of the trial (P < 0.05), while no differences were observed in the mean LnSCC of cows treated with CS (Fig. 6).

### Cytokine concentrations in milk

**Figure 7:**
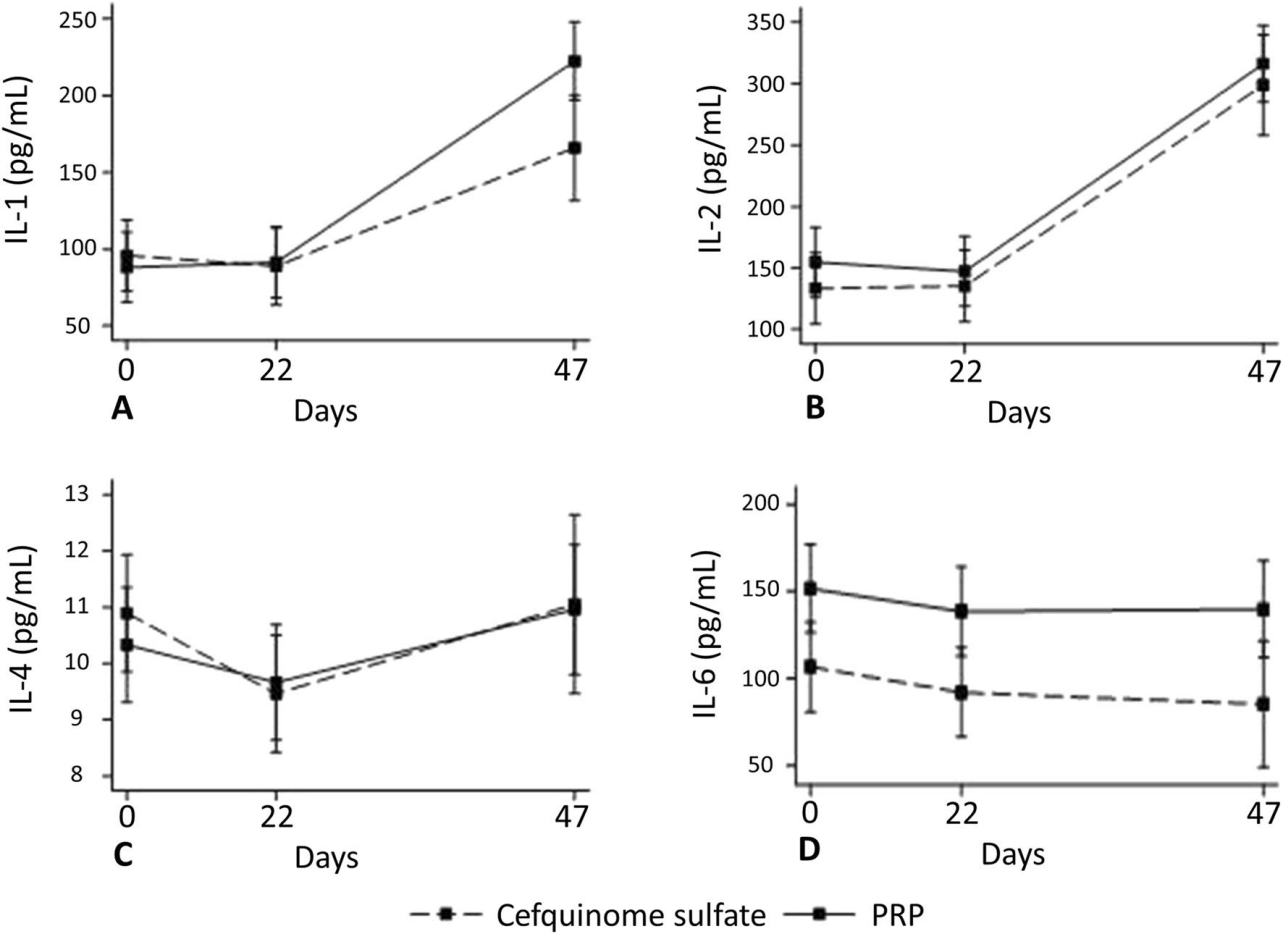
Mean concentration (95% CI) of milk interleukin 1 (IL-1) (**A**), IL-2 (**B**), IL-4 (**C**), and IL-6 (**D**) of cows with subclinical mastitis intramammary treated with 10 mL of P-PRP (pure platelet rich plasma) or 75 mg of CS (cefquinome sulfate) over three consecutive milkings. Zero represents the day of the treatment. Day 47 corresponds to cows that were treated twice, as they were refractory to the first treatment.

The concentrations of milk cytokines were only measured at day 0 and 21 in cows treated once, and at day 0, 21, and 47 in cows treated twice. The linear regression models showed a significant interaction between time and treatment for IL-1 (P = 0.008), IL-2 (P < 0.001), and IL-6 (P = 0.014) (Fig. 7A,B,D, respectively), while no differences were observed between time and treatment (P-PRP vs. CS) for IL-4 (P = 0.098) (Fig. 7C). Interestingly, IL-1 and IL-2 increased in cows that were treated twice, with the milk concentration of these cytokines being highest at day 47 (Fig. 7A,B).

**Figure 8:**
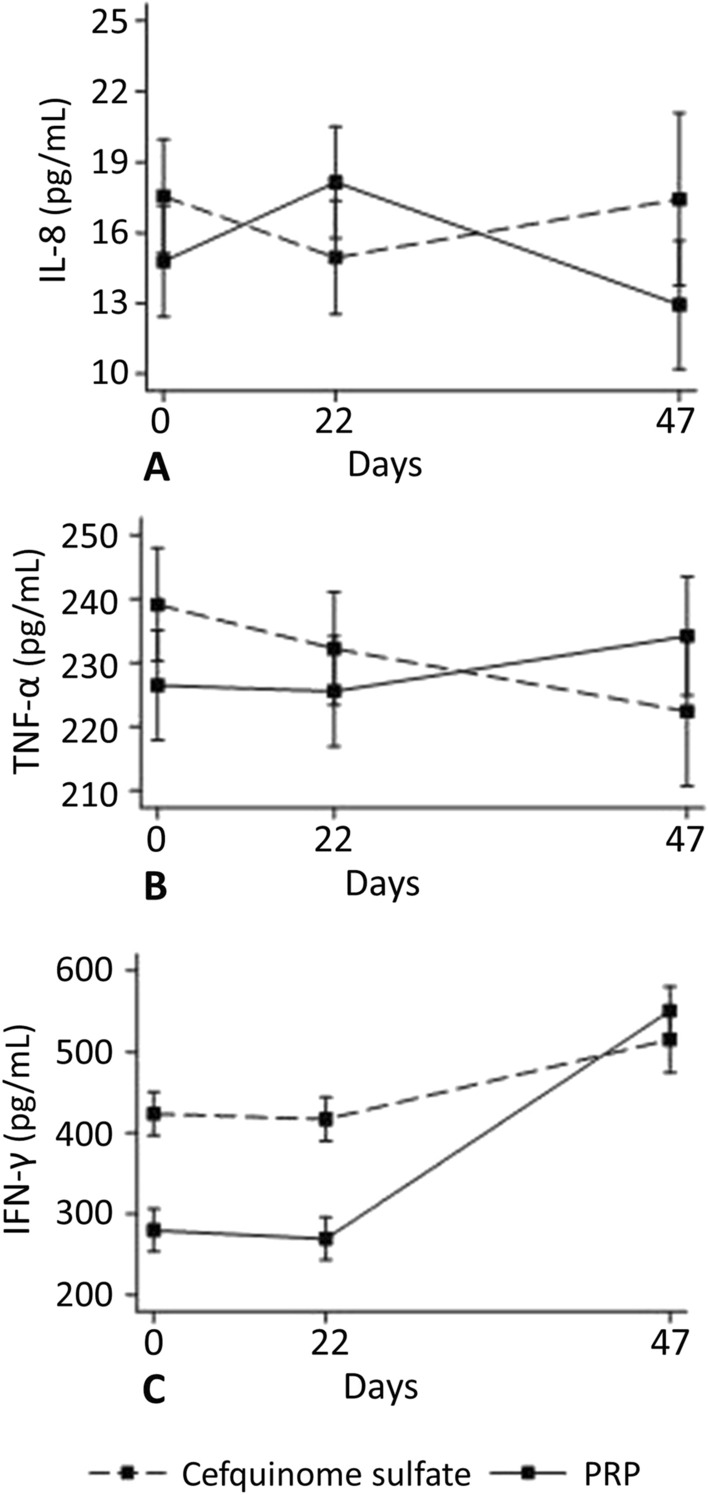
Mean concentration (95% CI) of milk interleukin 8 (IL-8) (**A**), interferon-γ (IFN-γ) (**B**), and tumor necrosis factor-α (TNF-α) (**C**) of cows with subclinical mastitis that received intramammary treatment with 10 mL of P-PRP (pure platelet rich plasma) or 75 mg of CS (cefquinome sulfate) over three consecutive milkings. Zero represents the day of the treatment. Day 47 corresponds to cows treated twice, as they were refractory to the first treatment.

Regarding the milk concentration of IL-8, IFN-γ, and TNF-α, the interaction between time and treatment (P-PRP vs. CS) was significant for these three cytokines (P ≤ 0.01) (Fig. 8A–C). The concentration of IFN-γ was higher in the P-PRP group compared to the CS group on days 0 and 21 (P < 0.001); however, it increased in both groups up to day 47 in cows treated twice (Fig. 8C), though no differences were observed between groups on day 47 (P = 0.16).

## Discussion

This study presents novel and complementary information regarding the clinical use of platelet-related hemoderivatives as a potential treatment for bovine mastitis^31^. However, from a comparative perspective our study differs from that of Lange-Consiglio et al.^31^ because we used P-PRP that was activated with calcium gluconate prior to its clinical use, whereas they used a platelet lysate derived from a PC that was frozen-and-thawed for inducing polypeptide release by platelet damage produced by temperature changes. This aspect is important since we used live cells contained in P-PRP that gradually released polypeptides (GFs and cytokines), whereas only polypeptides were apparently conserved for the treatment of the cows with mastitis in the aforementioned study^31^.

Another unique aspect of our research in comparison to Lange-Consiglio et al.^31^ is that we only evaluated cows with SCM caused by Gram-positives, whereas they evaluated cows with CM caused by both Gram-positive and Gram-negative. Unfortunately, only decrease of SCC was considered in the outcome, and the bacterial cure was not considered in Lange-Consiglio et al.’s^31^ study, and this fact prevents the comparison of the outcomes of both studies; however, we could compare the final SCC values of both studies.

The allogeneic platelet-related hemoderivative evaluated in this study was classified as a P-PRP that is characterized as plasma with a low concentration of platelets and a negligible concentration of leukocytes^15^. Notably, we obtained a hemoderivative with a lower concentration of platelets in comparison to the PC used by Lange-Consiglio et al.^31^ to prepare the platelet lysate employed in their study. However, it appears that both platelet-related hemoderivatives had some antibacterial effects against bovine mastitis-causing pathogens.

At this point, it is necessary to consider that the initial definition of PRP in the field of the human maxillofacial surgery, included 5 mL of plasma with a platelet concentration of 1000 platelets × 10^3^/µL^37^. This definition of PRP was also considered correct by other researchers^42^ for 14 years until new evidence revealed that some hemoderivatives with a lesser concentration of platelets demonstrated clinical usefulness^43^. Currently, both PRP definition and classification in humans continues to be controversial^44,45^. However, there is some consensus on PCs being classified according to the type and quantity of concentrated cells (platelets and leukocytes) and the substance used for activation, while a broad range of PCs can be considered PRPs^45–47^. In this sense, platelet concentration in a particular PRP can range from much lower, equal to, or several-fold greater than the platelet counts in whole blood, while the same could occur for the concentration of leukocytes in this substance^46^. Thus, our hemoderivative evaluated can be considered as a type of PRP, although the concentration of platelets in it was slightly similar to the platelets of whole blood from cows used for PC procurement in the present study.

We demonstrated that P-PRP has the capacity to gradually release TGF-β_1_ and PF4, because these polypeptides were only released in 40–50% of their platelets when compared to the release of the same mediators from platelet lysates (positive control). Moreover, it appears that calcium gluconate induces a massive release of PDGF-BB in the P-PRP, though this substance could possibly denature plasma complement C3, which could indicate that P-PRP should be used without calcium salts as activators to avoid denaturing plasma proteins with antibacterial activity.

We used allogeneic P-PRP because it is cumbersome to obtain the blood of the same cow in the field that will be treated with P-PRP due to SCM. Furthermore, it is important to consider that treating several cows in a mastitis control program is expected; thus, a high quantity of P-PRP could be necessary to treat several cows with SCM in a herd. Consequently, bovine practitioners can schedule the exact production of allogeneic P-PRP upon determining the number of affected cows in a herd. Furthermore, we employed heifers from a creole Colombian bovine breed because we observed in previous studies that young mares from equine creole breeds had higher growth factor concentration in PRP than horses from European breeds^48^. However, this assumption is only a hypothesis, and further studies are required to establish if a similar effect occurs with platelet-related hemoderivatives in bovines.

The CS antimicrobial was selected for the study because is a conventional broad-spectrum fourth-generation cephalosporin that is commonly used for the intramammary treatment of bovine mastitis caused by major mastitis-causing pathogens with an acceptable 65% cure risk^49^. That remains in high concentrations for up to 96 h regardless milk yield and udder health status^50^.

The CS in our study performed in accordance with the expected cure risk. However, P-PRP was lower than the findings described before by Italian researchers for a different but related hemocomponent (platelet lysate)^31^. The last could be related with the analysis, Italian study declare a success of the treatment based on the decrease of the linear score of SCC, but it does not include bacteriological cure. Besides, antimicrobial effect of P-PRP against Gram positive bacteria, including methicillin resistant *Staphylococcus aureus* has been described before in vitro*,* the limited effect of P-PRP could be affected by several factors not included in this study, such as the dilutional effect in mammary gland or even the short time of the evaluation of the biological product (PRP), because the potential mammary gland immunomodulation produced by this hemocomponent could take more time for bacteriological cure than when an antibiotic is used. However, this is only a hypothesis that should be evaluated in future studies.

According to research from New Zeeland, the hazard of retreatment for mastitis caused by the same pathogen within 30 d after first treatment could reach 20% according to the antimicrobial product^51^. Therefore, the treatment of all refractory cases was considered in our study. The last, also supported by findings that suggest a 53% of cure risk for retreatment of refractory mastitis cases where *Streptococcus agalactiae* was isolated^11^, similar to the CS group cure risk at retreatment. These results could be related to pathogen factors such as long-standing infections associated with high SCC, the pathogenicity and virulence factors of the strains and the resistance to the antimicrobial selected for the study^11,52^.

Regarding the overall bacteriological cure, 71% of the cows treated with CS and 30% of cows treated with P-PRP were negative to the bacteriological culture by the end of the study. Clearly, the CS exhibited a better antimicrobial response than the P-PRP in all cases according to the logistic models and overall results of the non-inferiority trial. Nevertheless, *Streptococcus* spp. group in the non-inferiority analysis was inconclusive, the last, could be related with the sample size. In this study, the sample size was calculated according to previous reports of CM cure risk, and the bacteria specie was not considered. In that order, the number of cows could not be enough to demonstrate the potential effect at individual bacterial level, and future research could elucidate the effect over specific bacteria.

In the present study, we observed that the overall LnSCC increased significantly in the group of cows treated with P-PRP at 21, 22, and 47 days when compared with the CS group. These results could be explained since a higher number of cows remained with IMI in the P-PRP group when compared to the CS group, and because P-PRP has chemotactic polypeptides, such as PDGFs^53^ and IL-8. Thus, further studies remain necessary to characterize the type and proportion of somatic cells stimulated during P-PRP treatment, and if these cells were related either to the IMI per se or a regenerative process in the mammary gland, the latter due to the contradictory effects presented in the Italian study (Lange-Consiglio et al., 2014), in which the infusion with the PC treatment resulted in an improvement in (decrease) of the SCC.

The cytokine arrangement (IL-1, 2, 4, 6, and 8, TNF-α and IFN-γ) measured in the milk of the cows of this study could represent the most common mediators in bovine mastitis studies^54,55^. In general, IL-1, 2, 6, and 8 and TNF-α are pro-inflammatory mediators, whereas IL-4 is an anti-inflammatory polypeptide and IFN-γ acts as a regulatory cytokine^56^. Generally, the milk concentrations of cytokines measured in this study were not influenced only by the treatment. Certain trends were only observed at specific time-points related to the concentration of some cytokines in the P-PRP group (i.e., IL-1 at 48 day, IL-6 at 22 and 47 days, IL-8 at 22 and 47 days, TNF-α at 47 days, and IFN-γ at 22 and 47 days). In considering these trends, it appears that the cows from both groups that improved with the first treatment had a lower degree of mammary gland damage than those cows that received an additional treatment. Thus, it is possible that milk cytokine screening be useful to classify SCM cows with udder damage and to establish outcomes when these animals are treated with antibiotics or PRP. However, this is merely a hypothesis, and further studies are required to support or reject it. In addition, the use of P-PRP could be limited by the temporary increase in SCC, which can affect the price of milk, and by the low risk of cure under the experimental conditions described in this study.

The present study had several limitations, including budget limitations, which should be considered as a base for future research in this field. Among these limitations we can numerate the following: (1) in the present study was not include an experimental group evaluating the effect of the combination of P-PRP plus antimicrobial. To note, this experimental group was not considered due to budget limitations and by the fact that for us was a priority to compare in isolated fashion the therapeutic effect of P-PRP against one approved effective antimicrobial available for mastitis treatment. (2) it was desirable to have included a group of healthy cows receiving the intramammary injection of P-PRP for better estimation of SCC and cytokine response by P-PRP in those cows with SCM and (3) also, the absence of a control group of SCM cows that did not receive any treatment was a limitation for this study, because without that group it was not possible to determine the spontaneous cure risk. The last occurred because the owners of the herds were not interested in leaving their cows without any treatment. Furthermore, the ethical committee of the Faculty of Agricultural Sciences of Universidad de Caldas, recommended using or considering the cows treated with antibiotics as a control group based on animal welfare concerns.

For further studies a control group and larger groups of cows with SCM caused by specific major pathogens should be included to improve the power of the analyses. Other conditions directly related to PRP treatment should also be evaluated in the future, such as increase or decrease the intramammary infusion volume of PRP, the platelet and leukocyte concentrations, and treatment schedules, among other factors.

## Conclusions

The group of SCM cows treated with PRP presented a lower rate of bacteriologic cure when compared to SCM animals treated with CS*.* In addition, our analysis did not confirm the efficacy of PRP. Both milk LnSCC and cytokines remain higher in SCM cows treated with PRP compared to animals treated with CS. These findings could be related to the cows that remains non-cured in the PRP group and the release of regulatory and chemotactic mediators (i.e., TGF-β_1_ and PDGFs) from activated PRP.

## Data Availability

All data supporting our findings are included in the manuscript. However, if readers need additional data of this study, these will be provided by the corresponding author carmona@ucaldas.edu.co.
